# Optimizing Workflow, Safety and Children’s Comfort in the Operating Theatre: A Mixed-Method Study Exploring Nurses’ and Caregivers’ Experiences and Possible Areas for Improvement

**DOI:** 10.3390/children13040528

**Published:** 2026-04-10

**Authors:** Raffaella Dobrina, Silvana Schreiber, Andrea Cassone, Paola Di Rocco, Valentina Cvetkovic, Martina Debelli, Giulia Galvani, Lucio Torelli, Loreto Lancia, Angelo Dante, Benedetta Tagliapietra

**Affiliations:** 1 Institute for Maternal and Child Health—IRCCS “Burlo Garofolo”, 34137 Trieste, Italy; 2 Clinical Department of Medical, Surgical and Health Sciences, University of Trieste, 34149 Trieste, Italy; torelli@units.it; 3 Department of Clinical Medicine, Public Health and Life and Environmental Sciences, University of L’Aquila, 67010 L’Aquila, Italy

**Keywords:** anesthesia induction, caregiver education, child comfort, nursing, pediatric surgery

## Abstract

**Highlights:**

**What are the main findings?**

- Caregivers rated their OT experience more positively than nurses did.
- Late education in the OT limits caregivers’ ability to support children.

**What are the implications of the main findings?**

- Early, clear information helps caregivers reduce child anxiety during induction.
- Calm, informed caregivers improve safety and workflow in the OT.

**Abstract:**

**Background/Objectives:** Few studies focus on essential information and training for responsible caregivers in the operating theatre (OT). Our study explored challenges, opportunities and critical information for caregivers close to the child until induction, drawing on the experiences of nurses and caregivers. **Methods**: A mixed-method exploratory sequential design was adopted in the OT and surgery wards of a maternal and child health hospital in Italy (2021–2023). **Results**: Twelve nurses were involved in 2 focus groups. The content analysis yielded 3 themes and 6 subthemes. Two questionnaires were developed for nurses and caregivers based on qualitative findings. The quantitative phase included 25 nurses and 140 caregivers. Results from the quantitative strand confirm findings from the qualitative strand. However, discrepancies in information needs highlight gaps. **Conclusions**: Optimizing family comfort and workflows in the OT depends on caregivers’ awareness of their role near the child, emphasizing mindful presence and awareness of their body movements—watching, touching and fidgeting.

## 1. Introduction

Worries, fears, and anxieties associated with the perioperative process, the operating theatre (OT) environment, anesthesia, and the possibility of suffering are common among both parents and young patients [1,2]. Such distress can compromise children’s cooperation and safety during anesthesia induction [3]. Multiple factors influence the family experience and the optimal organization of the OT, particularly in the preoperative phase.

A recent meta-analysis has highlighted that allowing parental presence in the OT until anesthesia induction can effectively reduce anxiety in both caregivers and children [2]. However, a systematic review suggests that caregivers do not always alleviate the child’s distress; in some cases, their own anxiety may heighten the child’s emotional response, thereby complicating the induction process [4]. This may stem from a lack of adequate information and involvement in the perioperative process or from caregivers’ limited ability to cope with the surgical experience [3,5]. Bandura’s Self-Efficacy Theory [6] offers a valuable framework for understanding how individuals’ beliefs in their own capabilities shape their preparedness, stress management, and engagement in high-pressure healthcare situations. In perioperative settings, lower caregiver self-efficacy may therefore contribute to increased anxiety and a diminished capacity to emotionally support the child, potentially making anesthesia induction more challenging.

These difficulties are further influenced by the increasing pressure of shorter hospital stays and widespread staff shortages, which limit the time available for professionals to adequately inform and involve children and caregivers, particularly given their varying levels of health literacy [7]. Various studies have demonstrated the efficacy of digital tools such as informational videos and virtual reality tours of the OT, in enhancing preoperative education [8,9]. Nevertheless, some healthcare ecosystems still face significant cultural and resource-based barriers that hinder the integration of these tools into routine clinical practice [10].

Moreover, to the best of our knowledge, few studies have explored the key factors and types of information that could ease OT nurses’ workflows while also improving the experience of families, particularly when these insights are grounded in the lived experiences of nurses and caregivers themselves. More specifically, limited attention has been paid to caregivers’ role behaviors within the OT, their impact on workflow efficiency and safety, and caregivers’ awareness of their informational needs and roles, as well as the alignment between caregivers’ self-perceptions and nurses’ expectations regarding their involvement in the OT. This study aims to fill this gap, focusing on a maternal and child health hospital where OT nurses raised key questions: “What are the benefits and challenges of caregiver presence in the OT during anesthesia induction?”; “What aspects of caregiver presence could be improved to better support the emotional well-being of children and ease staff workflow?”; “What critical information should be provided to caregivers of children undergoing surgery?”, and “How can caregiver presence in the OT be optimized to enhance staff workflow efficiency while maintaining the safety of the OT environment?”

This study explores the experiences of both nurses and caregivers when caregivers accompany children into the OT until anesthesia induction. Its overarching aim is to identify the key information and strategies that can be implemented to improve the comfort and well-being of patients and caregivers while simultaneously optimizing workflow in the OT during anesthesia induction. To achieve this, this study pursued the following specific aims:

(1) To qualitatively explore operating theatre nurses’ experiences of caregiver presence during anesthesia induction, including perceived benefits, challenges, and informational needs;
(2) To develop quantitative instruments based on qualitative findings;
(3) To quantitatively assess both nurses’ and caregivers’ perceptions of caregiver presence in the OT, exploring differences in perceived experiences, informational needs, and competencies in supporting the child, with the aim of identifying key areas for improvement in caregiver involvement and OT workflow.

## 2. Materials and Methods

### 2.1. Study Design

**Figure 1:**
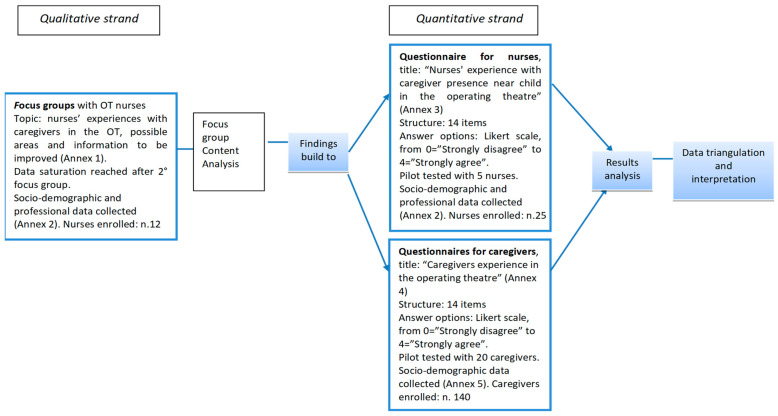
The steps of the exploratory sequential design and details of data collection.

To address the study aims, a mixed-method sequential design (QUAL → QUANT) was adopted to provide a more comprehensive understanding of the phenomenon, as the integration of qualitative and quantitative approaches allows both in-depth exploration and broader generalization of findings [11]. Among mixed-methods approaches, an exploratory sequential design was selected, in which qualitative and quantitative components were considered of equal importance. This design involves an initial qualitative phase followed by a quantitative phase, with integration, including triangulation of findings, occurring across the different stages of this study. The exploratory sequential design began with the collection and analysis of qualitative data. The focus group method was chosen for the qualitative portion of the data collection, as it has been shown to be effective in exploring complex phenomena, such as nurses’ experiences of having caregivers in the OT near the child [12]. Then, mixing strategies were applied to develop questionnaires for the collection of data of the quantitative strand, which were used to understand whether the previously collected data could be generalized to a larger sample. In this study, it was decided that in the quantitative strand, the contribution of both nurses and caregivers in reporting their experiences was of utmost importance to provide a more comprehensive understanding of the research topics from multiple angles. Finally, the results of the quantitative strand were triangulated with the findings from the qualitative strand and interpreted to mutually corroborate each other and answer all research questions. In particular, qualitative findings informed the development of the questionnaires and were also used to contextualize and interpret the quantitative results. The stages of study design are reported in Figure 1.

### 2.2. Setting

This study was conducted in a 136-bed maternal and child health hospital in north-eastern Italy. In particular, this study was carried out in the OT unit and in the ordinary surgery (21 beds) and day surgery (12 beds) wards. In 2025, the hospital performed approximately 2760 elective pediatric surgical procedures, corresponding to a mean of 11 procedures per day. The OT unit is staffed by a team of 25 nurses, 3 nurse aides, a head nurse and surgeons of different specialties (orthopedics, ophthalmology, dentistry, otorhinolaryngology, gynecology). Staff work in five OTs, the OT reception room, and the awakening room.

A general explanation of the perioperative process is given to the family on the day of the preoperative consultations. In this hospital, since the year 2000, caregivers are welcomed to accompany the child to the OT. Information regarding the OT and its equipment, sterile zones and dressing up is given when the family members arrive in the OT reception room. At the time of this study, there was no other approach to family education than oral explanations and pamphlets. Pamphlets contained information on preoperative hygiene preparation for the child, guidance on how to reassure and calm the child before surgery, and practical instructions on what to bring to the hospital (e.g., pajamas and personal items). The content of these materials was standardized and routinely provided for all surgical procedures. Before heading to the operating theatre, all children are given midazolam tranquilizer drops.

### 2.3. Data Collection

#### 2.3.1. Qualitative Strand

Focus groups were conducted in October 2021 by an expert moderator. An observer was present to take field notes. The sessions took place in a room reserved for staff in the OT unit and were digitally recorded. The topic of discussion was nurses’ experiences with caregivers in the OT, and possible areas and information to be improved. The interview guide is available in Supplementary File S1. A pilot interview was conducted with two nurses to evaluate the clarity of the questions and their alignment with this study’s objectives. The nurses reported no issues, and the focus groups were subsequently conducted. Socio-demographic and professional data were also collected (data collection sheet available in Supplementary File S2). Data saturation was reached after the second focus group.

#### 2.3.2. Quantitative Strand

Following the steps of the exploratory sequential design, a questionnaire was developed for the quantitative strand of data collection to explore nurses’ experiences in the OT with a caregiver present close to the child. The qualitative findings were used to formulate a pool of items by 3 researchers. Two nurses from the surgical ward and the OT head nurse were asked to evaluate the items according to wording, allocation, and the scores on the Likert scale. Some items were revised to improve understanding. The questionnaire was pilot tested with five nurses to assess the relevance, clarity and comprehensibility of the items using an open-ended question. As there were no comments, the questionnaire was ready to be distributed to a larger sample of theatre nurses, who were all asked to participate and give their responses based on their general perception of caregivers’ presence in the OT. The final questionnaire consisted of 14 items, each rated on a 5-point Likert scale ranging from 1 (strongly disagree) to 5 (strongly agree). The questionnaire is available in Supplementary File S3. Then, another questionnaire (Supplementary File S4) was developed by the research team, mirroring the 14 items administered to nurses but adapted to capture the caregiver perspective. For example, the nurses’ item 2 “In the operating theatre the caregiver was anxious or agitated” was rephrased for caregivers as “In the operating theatre you felt anxious or agitated”. This second questionnaire was pilot tested with 20 caregivers and since no caregiver left comments, it was distributed to a larger sample. Sample size for caregivers was calculated: assuming a proportion of positive experience (answers “4” or “5”) in the OT equal to 90% with a precision of 5% and alpha = 0.05, 139 subjects were needed to reach sample size. This assumption was pragmatic, reflecting the expectation of high satisfaction levels in this context, although it was not based on specific prior empirical data. Sociodemographic data were also collected from caregivers (data collection sheet available in Supplementary File S5).

Quantitative data collection was performed from June 2022 to March 2023.

Details of data collection for the qualitative and quantitative strands are described in Figure 1.

### 2.4. Participants

#### 2.4.1. Qualitative Strand

A purposive sampling method was used to enroll nurses from the OT who had more than six months of experience, excluding those who did not provide direct patient care.

#### 2.4.2. Quantitative Strand

A convenience sampling method was used to recruit caregivers of children (0–17 years old) who underwent any kind of surgery and attended the surgery or day surgery ward, who were approached approximately two hours after the child’s return to the ward following surgery. Caregivers were eligible if they accompanied their child in the OT and declared that they had no difficulty in reading and understanding Italian. Caregivers with visual or hearing disabilities that could impair data collection or informed consent for participation in this study were excluded. For the quantitative phase, the eligibility criteria for nurses were the same as those for the qualitative phase.

### 2.5. Ethics

Ethical approval was obtained from an Institutional Review Board (IRB-BURLO 08/2021 1 September 2021). Potential participants, a few hours after their child returned from surgery, received information about this study and an information sheet explaining its objectives and processes. A member of the research team remained available in case potential participants needed further clarification. Participation in this study was on a voluntary basis and informed consent was obtained from each participant. The anonymity of the participants was guaranteed.

### 2.6. Data Analysis

#### 2.6.1. Qualitative Data Analysis

Data were treated and processed anonymously. The audio recordings of the focus groups were transcribed verbatim. Then, the transcripts were inserted into the right-hand side of a three-column table in a word processor file, keeping the middle column for comments and the left column for themes. Three researchers (RD, BT, VC) performed an inductive content analysis process [12], first independently and then by consensus.

The researchers first read and re-read the transcripts to familiarize themselves with the data and identify units of meaning. Then, they performed manual coding. After analytical reflection, the first draft of sub-themes and themes was prepared and discussed among the researchers until a final agreement was reached.

To ensure the trustworthiness of the qualitative analysis, a series of complementary strategies were employed. First, researchers meticulously and transparently detailed the procedures, methods, and settings. Focus groups were facilitated by the same expert researcher using a purposive sample of participants. Field notes were utilized to support debriefing and data analysis. Personal assumptions were shared among the researchers before the qualitative data analysis, and rigorous bracketing and triangulation processes were employed to prevent bias, maintain objectivity, and focus on participants’ experiences. To support credibility, quotations were extracted for each sub-theme.

#### 2.6.2. Quantitative Data Analysis

Quantitative data from the two questionnaires and the sociodemographic and professional questionnaires were inserted into the web-based software platform REDCap (Research Electronic Data Capture 4.0.17—© 2024 Vanderbilt University) and managed through REDCap electronic data capture tools. For data analysis, the software R Core Team (2021) was used. Frequency and percentage distribution or median and interquartile range (or mean and standard deviation) were calculated to describe the demographic and professional variables and data from the two questionnaires responses. Since, to our knowledge, there is no recognized universal classification of complexity in surgery, the type of surgery the children underwent according to the caregivers was divided into low complexity (1) and medium to high complexity (2). If the caregivers did not indicate a specific type of surgery, such as “orthopedic” in general, it was not classified. Low complexity procedures included quick, minimally invasive procedures that are usually performed on a day surgery basis or with a maximum of one night hospitalization. Examples: adenotonsillectomy, circumcision, removal of foot screws, removal of a chalazion. Medium to high complexity procedures included all other surgeries including hypospadias, rhinoplasty, pyeloureteroplasty, rectal reconstruction, and arthrodesis (surgery type classification available in Supplementary File S6).

Non-parametric Mann–Whitney or Kruskal–Wallis tests were conducted to examine correlations between responses to different questionnaire items, as well as between questionnaire responses and variables such as caregivers’ gender, age, education, health professional status, and the child’s surgery classification.

## 3. Results

### 3.1. Qualitative Strand Results

Twelve nurses (46.15% of nursing staff) from the OT participated in 2 focus groups (FG), (six per group). The average age of the participants was 45 years, 75% of whom were female. Socio-demographic characteristics of participants are presented in Table 1.

#### 3.1.1. Theme 1. Mindful Presence of Caregivers in the Operating Theatre

The presence of a parent is identified as a positive and crucial element by all participants. Some older participants recall a time when parents were not permitted in the OT, highlighting the significance of this change. This theme highlights that when caregivers understand their role, remain calm, and when children’s preferences are respected, their presence enhances the child’s comfort and supports a safer induction process. However, many parents remain unaware of the critical role of their mindful presence in calming their child and ensuring the safety of procedures in the OT, including aspects such as directing their attention towards the child rather than medical equipment, avoiding contact with sterile areas, and minimizing restless movements such as pacing or fidgeting.

#### 3.1.2. Subtheme 1. Supporting the Child’s Needs

Participants perceived caregivers as valuable in supporting the child by conveying security, serenity, and trust before surgery. They also assist in pre-anesthesia procedures, such as distracting the child during interventions involving needles. However, participants noted that caregivers often focus on monitoring healthcare professionals’ actions, believing it gives them a sense of control over the situation. Instead, this behavior tends to heighten their stress and, in turn, transfer anxiety to the child. One participant explained:

“While we are inserting venous access, the parent’s role is to engage the child through play to distract them. But if they watch what we are doing, their child will also watch”.

(FG1)

Therefore, participants agreed that caregivers’ presence is most beneficial when they understand their role in providing emotional support and distraction while remaining calm. The discussion also highlighted the importance of listening to patients’ needs and respecting their preferences, such as an adolescent’s choice not to have their mother present in the OT. Many participants reported that they actively ask children about their preferences and explain to parents why respecting these choices is crucial for the child’s comfort. One participant stated:

“…I ask the kid if he wants his mother to accompany him or not. If he prefers to go on his own, it is important that his mother respects his wishes…many mothers agitate their children, and they seem to be aware of this.”.

(FG1)

#### 3.1.3. Subtheme 2. Compliance with Safety Standards

Participants emphasized that the presence of a caregiver in the OT plays a crucial role in ensuring the completeness of surgical and medical records. Upon arrival at the OT reception room, the operating checklist is reviewed, and parental presence helps streamline this process, reducing the risk of surgical delays. Additionally, caregivers contribute to their child’s cooperation with OT nurses, thereby enhancing procedural safety.

However, participants also expressed concerns about caregivers who appear disoriented and uncoordinated in the OT environment, potentially compromising safety. One participant remarked:

“Some parents arrive in the OT looking completely lost… I need them to be collaborative and not touch anything! There are clean and sterile areas that must be preserved”.

(FG2)

#### 3.1.4. Theme 2: Critical Issues in the Involvement of Caregivers and Children

This theme underscores the crucial issues raised by multiple participants during the focus groups discussion about the lack of caregivers’ preparation for the OT environment and procedures which often leads to confusion and the need for additional nursing resources. In particular, when caregivers are not equipped to prepare their children for surgery, children frequently arrive distressed and unprepared, further complicating induction and increasing emotional discomfort.

#### 3.1.5. Subtheme 1. Critical Issues in Engaging Caregivers

Several participants agreed that caregivers often exhibit significant information deficits upon entering the OT. For instance, nurses reported that some caregivers arrive unaware that they are allowed to accompany their child into the OT and remain until the child falls asleep. Additionally, caregivers who are fearful of anesthesia procedures struggle to provide effective support for their child, often requiring additional nursing resources. One participant reported:

“When the parents are in a situation where they feel disoriented… let’s say… this creates difficulties for us nurses, who have to take care not only of the child but also of the parents.”.

(FG2)

Participants noted that, beyond the lack of adequate information about the OT environment and procedures, individual personality traits also contribute to caregivers’ agitation. One participant remarked:

“Sometimes, it’s just the parent’s anxious nature… We see them coming, and we already know how the induction will go”.

(FG1)

Additionally, participants recognized that providing caregivers with comprehensive information only upon arrival at the OT is ineffective, as this moment is highly stressful, making it difficult for them to absorb critical details. One participant emphasized:

“The OT is an unsuitable place to provide education. Preoperative consultations do not include an OT nurse, but we should be there”.

(FG2)

Participants regretted that, due to organizational constraints, OT nurses are currently unable to participate in preoperative consultations to adequately prepare caregivers for the OT experience.

#### 3.1.6. Subtheme 2. Critical Issues in Engaging Children

Participants reported that when they find attentive caregivers who are supportive and who inform their children well, the children cope very well and face the journey very calmly. However, it emerged from all participants that they perceive a serious lack of information on the part of many young patients. The latter are very often unaware of what they are about to encounter, which invariably causes the child to become very agitated for the fear of the unknown such as the new and unfamiliar environment of the OT, the special equipment, the presence of so many health workers they have never seen before, and the new sounds and noises. One participant reported:

“Many parents do not explain anything to their children about surgery or the OT (…) Maybe because they are afraid of frightening the child or do not feel able to deal with the child’s possible reactions”.

(FG1)

Participants reported that caregivers tend to hide the truth about surgery and all the steps leading up to it, probably in an attempt to protect their children or because they do not know how to handle possible reactions. One participant reported:

“It seems incredible, but some children arrive not informed about their surgery. I had a parent who came to the OT and told the child that he came here to take pictures!”.

(FG1)

#### 3.1.7. Theme 3. Education to Improve the Presence of Children and Caregivers in the OT

Participants were eager to suggest strategies for improving caregiver involvement and proposed various educational approaches aimed at improving children’s perioperative experience while easing the demands on nursing workflow.

#### 3.1.8. Subtheme 1. Education: Who and What

Participants unanimously agreed on the need to enhance educational efforts for both caregivers and children. They emphasized the importance of explaining OT safety rules and procedures before anesthesia using language tailored to different age groups. One participant highlighted the benefits of engaging children through exposure to their surroundings:

“Children are naturally curious… Showing them the equipment we use, cables, pulse oximeters, monitors, and involving them would help them feel more comfortable”.

(FG2)

Additionally, participants stressed the need to inform caregivers that their role goes beyond simply accompanying their child. Caregivers should understand their responsibility in supporting their child emotionally and assisting the healthcare team in ensuring safe and efficient procedures.

One participant suggested:

“We should spend more time helping parents understand the importance of their role and the need to inform their children”.

(FG1)

#### 3.1.9. Subtheme 2. Education: When and How

Participants agreed that the waiting time in the OT reception room, which can last up to 30 min, presents a valuable opportunity for nurses to interact with caregivers and patients while reinforcing essential information. However, they also acknowledged that this period is highly stressful, making it difficult for families to fully process new information. To address this, participants suggested that education should take place during preoperative consultations, using the OT reception room only to reiterate key details. However, they recognized the challenge of integrating OT nurses into preoperative consultations due to organizational limitations. One participant proposed an alternative approach:

“If we cannot be present at pre-admission consultations for organizational reasons, we could train the nurses conducting these consultations to provide caregivers with essential OT information in advance… BEFORE they arrive at the operating theatre”.

(FG1)

Participants strongly agreed that caregivers should receive information several days before surgery, allowing them time to process it. The time in the OT reception room should then focus only on reinforcing the most critical points. Participants also proposed using digital tools to enhance family education. One participant suggested:

“We could create educational videos ourselves”.

(FG2)

Another participant, recognizing hospital time constraints, proposed an interactive approach:

“We could organize digital platform meetings with groups of parents whose children are undergoing surgery… to save time”.

(FG1)

In this model, families would receive an access link to scheduled virtual meetings where an OT nurse would provide information, showcase the OT environment, and answer questions in real time. This flexible approach would allow caregivers to participate from home, ensuring they and their children are better prepared for the surgical experience.

### 3.2. Quantitative Strand Results

Twenty-five questionnaires (100% of total) were returned completed by OT nurses. Of the respondents, 88% were female and 64% held the title for more than 15 years (Table 1).

As for caregivers’ data collection, 140 questionnaires were completed, reaching the targeted sample size. Of these, 100% were parents, of whom 85% were mothers. Their children had an average age of 8.63 years (min 0–max 18), 26.4% of whom had previously received other surgery interventions. Other data are reported in Table 1. Data analysis revealed that the response percentages were evenly distributed across the various options in almost all items.

Questionnaire’s results on caregivers’ experience in the OT as perceived by caregivers and nurses are reported in Table 2. The two questionnaires contained the same statements, with wording adapted to reflect the different perspectives. For example, item 12 was phrased as “Caregivers know how long they can stay with the child in the operating theatre” for nurses and as “You knew how long you would be able to stay with your son/daughter in the operating theatre” for caregivers. A positive experience about the presence of caregivers on the OT close to the child was perceived by both nurses and caregivers, with better agreement between caregivers. Caregivers also reported better scores than nurses in the perception of their comfort, and competence in supporting the child than nurses. As for the information received, caregivers again reported better experience than nurses, e.g., the timing of the information received.

Nurses perceived that caregivers needed more information, and strategies to distract the child than caregivers. In addition, nurses perceived caregivers as more anxious, and more distracted by the OT environment and children to be more anxious/agitated than the caregivers perceived. The difference between nurses and caregivers’ responses was statistically significant in all items (*p*-value: <0.001).

**Figure 2:**
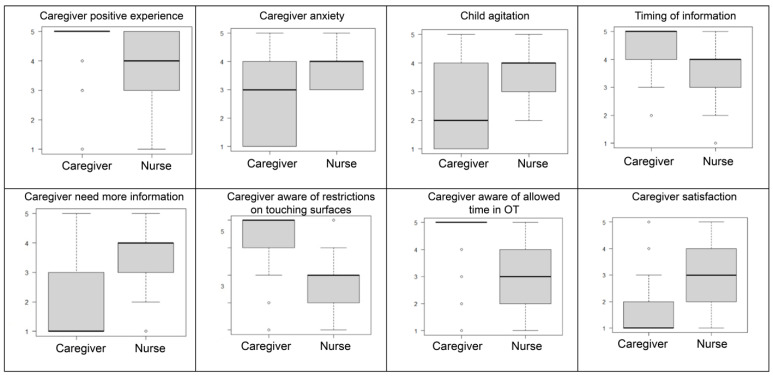
Examples of the distribution of responses of nurses and caregivers about experience in the OT.

Examples of the distribution of responses of nurses and caregivers are presented as boxplots in Figure 2.

In the correlation analysis between the answers of caregivers, a significant correlation emerged between parental anxiety and child agitation (*p* < 0.001). In the correlation analysis between the socio-demographic variables and the caregivers’ answers to the questionnaire, no significant correlation was found between gender, age, or being a health professional, and caregivers’ answers to the questionnaire. However, caregivers with a lower educational level (middle school) reported significantly higher levels of difficulty experienced in the OT (item 6 in the questionnaire on Supplementary File S4) compared to those with a university degree (*p* = 0.018). Moreover, a significant difference was found between child agitation and low complexity surgery (*p* = 0.013), with higher agitation reported for low complexity surgery.

## 4. Discussion

The mixed-method design adopted in this study offers a novel contribution to this topic providing a deeper understanding of the experiences and training opportunities of caregivers of children in the OT until anesthesia induction, as well as those of the nurses involved. The involvement of nurses and caregivers, together with the integration of qualitative and quantitative findings, enabled a more comprehensive understanding of the specific information gaps, challenges and opportunities to optimize comfort and safety of children and their caregiver from multiple perspectives.

In line with findings from the literature [13,14], nurses regard caregivers as a valuable resource when present in the OT prior to induction, as they can facilitate the sign-in workflow, help maintain their child’s calmness, and collaborate effectively with the anesthesia team during procedures. However, consistent with previous studies [3], nurses also noted that caregivers’ anxiety may inadvertently heighten children’s distress. Furthermore, the lack of preoperative information for both children and caregivers, together with caregivers’ limited awareness of appropriate behavior and responsibilities in the OT, emerged as key contributing factors. These issues not only increase children’s stress but may also disrupt the anesthetic process.

The heightened agitation reported in children undergoing low-complexity surgeries may stem from the relatively shorter time healthcare providers devote to family information and preparation. As suggested in previous research [7], more complex surgeries often involve more extensive discussions, emotional support, and information sharing, which may mitigate anxiety for both children and caregivers.

Caregiver’s education level also emerged as a relevant factor: individuals with lower education reported greater difficulties during their time in the OT. This aligns with evidence linking lower education to reduced health literacy, which can hinder the ability to process medical information and cope with stressful situations during surgery [7,15].

Nurses strongly agreed, across both qualitative and quantitative data, that preoperative information and caregiver engagement require improvement. These findings were in line with previous research [16,17]. They emphasized the need to explain OT rules and procedures to both children and caregivers in an age-appropriate and accessible manner. In particular, nurses emphasized the need for caregivers to be more aware of their body movements and interactions with the sterile environment in the OT, including their gaze direction and of their role in calming and distracting the child. All these aspects influence both child distraction and OT safety. Nurses stressed the importance of educating caregivers about their specific role in the OT. This role, as other authors suggested [16,17], should extend beyond mere presence, to a mindful active collaboration with the OT team to address the child’s needs and emotions and to support anesthetic procedures. To this end, nurses suggest organizational strategies such as identifying adequate times and places to ensure an effective transmission of information and education, which should then be only reinforced in the OT. Nurses also propose other solutions such as involving an OT nurse in preoperative consultations, training surgical ward nurses in parent OT education.

A notable finding of this study is the discrepancy between nurses’ and caregivers’ perceptions. Nurses reported that caregivers urgently required more information and strategies to effectively support their child and the OT team. In contrast, caregivers expressed a more positive view of their own roles and the child’s experience. Additionally, caregivers felt that the information provided about the OT was delivered in a timely and comprehensive manner, covering topics such as sterile environment protocols, dressing guidelines, and the allowed time with the child. These discrepancies highlight a gap in the care alliance: nurses appear more attuned to the importance of environmental sterility and distraction strategies, whereas caregivers may not fully recognize the relevance of these factors, even when information is provided adequately, overestimating their own knowledge and abilities [7]. This attitude is described in the literature as the Dunning–Kruger effect, indicating that individuals, typically with low health literacy, tend to overestimate their understanding of health-related issues [18]. However, caregivers’ confidence may also represent a valuable resource. According to Bandura’s Self-Efficacy Theory [6], high perceived self-efficacy supports initiation and persistence in challenging tasks, managing stress, and applying learned skills effectively. In the OT setting, caregivers’ self-efficacy likely influences their emotional regulation and their ability to support the child and collaborate with the surgical team, regardless of the objective adequacy of their preparation. Thus, professionals should reinforce caregivers’ sense of competence, as a strong belief in one’s own abilities can encourage the acquisition and application of new knowledge and skills [6,19].

These quantitative and qualitative findings highlight the need for targeted educational interventions, particularly regarding caregivers’ role in anxiety management and the use of distraction strategies. Caregivers’ confidence and self-efficacy may represent a valuable resource in this context, providing a favorable basis for the acquisition of appropriate knowledge and behaviors within the OT. In this context, nurses should aim to support parents’ agency while complementing it with targeted education where needed. Education programs could integrate anxiety management techniques and emphasize collaborative communication to ensure caregivers’ confidence aligns with their ability to effectively support their child and the OT team. As other authors have discussed, such engagement of caregivers in the OT care process would enhance their health literacy, empowerment, and satisfaction [14,20,21].

While the literature supports the effectiveness of educational and non-pharmacological interventions, such as virtual reality tools, in reducing children’s anxiety and improving caregiver involvement [8,9,22,23], evidence on caregiver education regarding behaviors related to sterility in the OT remains limited. Integrating these aspects into existing educational strategies, including digital and immersive tools such as virtual reality, may represent an important area for future research.

This study provides new insights into informational gaps in the preoperative process, highlighting differences between caregivers’ and nurses’ perceptions of preparation. It also emphasizes the importance of better supporting caregivers’ involvement through targeted education. In this context, it becomes essential for caregivers to recognize their role alongside the child, adopting a mindful presence and awareness of their behaviors in the OT, including where they direct their attention, what they touch, and their body movements, in order to prevent contamination and maintain the sterility of the environment.

### Strengths and Limitations

This is the first study that integrates multiple perspectives (both nurses and caregivers) and rigorously employs trustworthiness strategies in its mixed-methods design to optimize family education and organization around the induction of anesthesia.

However, this study has some limitations. First, this study adopted an observational design, which does not allow causal inferences. Additionally, the sample was recruited from a single center, potentially limiting generalizability. Moreover, data were based on self-reported measures, which may be subject to response and social desirability bias. Furthermore, the questionnaire for caregivers was designed for caregivers of children aged 0–17 years. To allow a more specific assessment of experiences, further research could refine the caregivers’ questionnaires based on children’s age groups, such as for caregivers of newborns, preschool children, children aged 6–11 or those aged 12–17 years. Moreover, bias related to caregivers’ emotional state after surgery may have impacted their reporting in questionnaires. In addition, the presence of participants who had previously received other interventions may represent a source of bias, as prior experience could have influenced caregivers’ perceptions and responses. Additionally, a proportion of caregivers were healthcare professionals, which may have further influenced their expectations and evaluations.

Furthermore, the eligibility criteria requiring caregivers to have adequate Italian literacy and no sensory impairments may have introduced selection bias, potentially excluding individuals with greater vulnerability.

Regarding the type of surgery, there may have been classification errors because this information could not be verified in order to preserve respondents’ anonymity.

Finally, the developed questionnaires have gone through content and face validity analysis. However, future research is needed to evaluate the psychometric properties of the survey on a larger sample to effectively explore these topics also in other settings.

## 5. Conclusions

Our study identifies critical information and training opportunities influencing family comfort, the safety and timing of the procedures until children anesthesia induction in the OT. This extends prior work in the literature by offering an integrated, multi-perspective understanding of these needs. Nurses in our study highlighted the importance of improving preoperative information and preparing caregivers for a mindful and active presence in the OT, starting from pre-admission day. Key elements include clear education on caregivers’ role in the OT at the child’s side and awareness of their body movements, gaze direction, and interactions with the sterile environment. In turn, findings from caregivers suggest that their personal confidence can be leveraged as a resource for effective training. These insights may support OT nurses and managers in optimizing organizational processes and tailoring educational approaches for families accessing the OT during the preoperative preparation.

In addition, the questionnaires developed in our study could undergo further validation to enable a wider applicability in other hospital settings.

Based on our findings, four concrete steps may be implemented to improve children’s comfort, OT workflow and safety:

(1) Delivering standardized OT education on the pre-admission day, including caregiver role coaching, sterile-zone rules, and child-distraction strategies;
(2) Involving an OT nurse in preoperative consultations, or, if it is not feasible, ensuring that OT nurses train ward nurses to provide accurate OT-specific education;
(3) Developing a digital learning package for caregivers, such as a checklist of “do’s and don’ts,” brief videos introducing the OT environment, explanations of sterile versus non-sterile areas, and short demonstrations of distraction techniques, to reinforce key messages
(4) Reinforcing these key messages in the OT reception area immediately before transfer to the OT.

## Figures and Tables

**Table 1:** Socio-demographic characteristics of participants.

	Focus Group Nurses (N = 12)	Questionnaire Nurses (N = 25)	Questionnaire Caregivers (N = 140)	Characteristics of Children
Gender	M: 3 (25%) F: 9 (75%)	M: 3 (12%) F: 22 (88%)	M: 21 (15%) F: 119 (85%) (100% parents)	M: 83 (59.3%) F: 57 (40.7%)
Age	<25: - 26–30: 2 (16.7%) 31–35: 2 (16.7%) 36–40: - 41–45: 1 (8.3%) 46–50: 2 (16.7%) 51–55: 2 (16.7%) >55: 3 (25%)	<25: 1 (4%) 26–30: 1 (4%) 31–35: 4 (16%) 36–40: 2 (8%) 41–45: 3 (12%) 46–50: 6 (24%) 51–55: 4 (16%) >55: 4 (16%)	<30: 7 (5%) 31–35: 23 (16.43%) 36–40: 33 (23.57%) 41–45: 29 (20.71%) 46–50: 30 (21.43%) >51: 18 (12.86%)	0–2: 14 (10%) 3–5: 32 (22.86%) 6–8: 21 (15%) 9–11: 21 (15%) 12–14: 40 (28.57%) >15: 12 (8.57%)
Surgery	-	-	-	Low complexity: 94 (67.1%) Medium to high complexity: 39 (27.8%) Not classified: 7 (5%)
Child’s Previous Surgeries				None: 103 (73.6%) One or more: 37 (26.4%)
Professional Qualification	Pediatric Nurse: 1 (8.3%) Nurse: 11 (91.6%)	Pediatric Nurse: 3 (12%) Nurse: 22 (88%)	Manager/Entrepreneur: 8 (5.7%) Freelancer: 14 (10%) Employee: 51 (36.4%) Workman: 18 (12.9%) Unemployed: 12 (8.6%) Other: 37 (26.4%) (Health Professional: 19–13.6%)	-
Years Since Graduation	<5: 4 (36.4%) 5–10: - 10–15: - 15–20: - >20: 7 (63.3%)	<5: 1 (4%) 5–10: 4 (16%) 10–15: 4 (16%) 15–20: 1 (4%) >20: 15 (60%)	-	-
Years Of Work At OT Unit	<5: 5 (41.7%) 5–10: - 10–15: 1 (8.3%) 15–20: 1 (8.3%) >20: 5 (41.7%)	<5: 10 (40%) 5–10: 4 (16%) 10–15: 6 (24%) 15–20: 2 (8%) >20: 3 (12%)	-	-
Country Of Origin	-	-	Italy: 122 (87.1%) Other: 18 (12.9%)	-
Degree	-	-	Middle school: 11 (7.9%) Upper Secondary Schools: 76 (54.3%) University: 28 (27.1%) Post-University: 12 (8.6%) Other: 3 (2%)	-

**Table 2:** Caregivers’ experiences and competencies in the OT perceived by nurses and caregivers themselves.

	Nurses				Caregivers				
	Mean	SD ^1^	Median	IQR ^1^	Mean	SD ^1^	Median	IQR ^1^	*p*-Value
Caregiver positive experience	3.84	1.11	4	3–5	4.73	0.59	5	5–5	<0.001
Caregiver anxiety	3.88	0.73	4	3–4	2.65	1.48	3	1–4	<0.001
Perceived child anxiety/agitation	3.80	0.87	4	3–4	2.73	1.46	2	1–4	<0.001
Timing of information provided to caregiver	3.56	1.12	4	3–4	4.54	0.77	5	4–5	<0.001
Caregiver need for more information	3.60	1.12	4	3–4	2.12	1.50	1	1–3	<0.001
Caregiver difficulty close to child	2.80	1.08	3	2–4	1.66	1.31	1	1–1.25	<0.001
Caregiver efficacy of support to child	3.84	0.85	4	3–4	4.6	0.77	5	4–5	<0.001
Caregiver comfort in OT	2.72	0.98	3	2–3	4.46	0.89	5	4–5	<0.001
Caregiver competence in supporting the child	2.76	0.92	3	2–3	4.36	0.85	5	4–5	<0.001
Caregiver needs more strategies to support child	3.84	1.11	4	3–5	2.07	1.37	1	1–3	<0.001
Caregiver distraction	2.92	1.15	3	2–4	1.67	1.24	1	1–2	<0.001
Caregiver aware of allowed time in OT	3	1.30	3	2–4	4.68	0.82	5	5–5	<0.001
Caregiver aware of restrictions on touching surfaces	2.60	1.15	3	2–3	4.24	1.19	5	4–5	<0.001
Caregiver aware of required dressing before entering OT	3.84	0.99	4	2–5	4.58	0.97	5	5–5	<0.001

^1^ Legend: SD: Standard deviation; IQR: Interquartile range.

## Data Availability

The original data supporting the conclusions of this article are openly available in Zenodo at 10.5281/zenodo.18834499.

## Supplementary Materials

The following supporting information can be downloaded at: https://www.mdpi.com/article/10.3390/children13040528/s1, Supplementary File S1. Focus group interview guide. Supplementary File S2. Sociodemographic for nurses. Supplementary File S3. Questionnaire nurses’ experiences. Supplementary File S4. Questionnaire caregiver’s experience. Supplementary File S5. Sociodemographic for caregivers. Supplementary File S6. Type of surgery.

## Abbreviations

The following abbreviations are used in this manuscript:

OT	Operating Theatre
FG	Focus Group
QUAL	Qualitative
QUANT	Quantitative
