# Supplementary material for: Optimizing Workflow, Safety and Children’s Comfort in the Operating Theatre: A Mixed-Method Study Exploring Nurses’ and Caregivers’ Experiences and Possible Areas for Improvement
Source: Children (Basel). 2026 Apr 10;13(4):528. doi: 10.3390/children13040528 (PMC13115178; doi:10.3390/children13040528)
Supplement: Supplementary file 1 [file children-13-00528-s001.zip › Supplementary file S1. Focus group interview guide.pdf]

Supplementary file S1. Focus group interview guide.

The interview guide of the focus groups with nurses included the following cues:

1. What is your experience regarding the presence of parents in the OT next to the child until anesthesia induction?
2. In your opinion, are there aspects that can be improved? Which ones?
3. What do you think is the critical information to be provided to caregivers of children accessing the OT and the best timing of providing such information in the perioperative process?
4. How could the presence of caregivers in the OT be made more effective for the peace of mind of the child, the caregiver and your work?
