# Supplementary material for: Optimizing Workflow, Safety and Children’s Comfort in the Operating Theatre: A Mixed-Method Study Exploring Nurses’ and Caregivers’ Experiences and Possible Areas for Improvement
Source: Children (Basel). 2026 Apr 10;13(4):528. doi: 10.3390/children13040528 (PMC13115178; doi:10.3390/children13040528)
Supplement: Supplementary file 1 [file children-13-00528-s001.zip › Supplementary file S2. Sociodemographic for nurses.pdf]

Supplementary file S2. SOCIODEMOGRAPHIC and PROFESSIONAL DATA COLLECTION SHEET FOR NURSES (in English and Italian)

SOCIODEMOGRAPHIC and PROFESSIONAL DATA COLLECTION SHEET FOR NURSES (in English)

1) Sex:

□ Male □ Female

2) Age:

□ < 25 □ from 26 to 30 □ from 31 to 35 □ from 36 to 40 □ from 41 to 45

□ from 46 to 50 □ from 51 to 55 □ > 55 years old

3) Professional qualification:

□ nurse □ paediatric nurse □ other (specify)_______________________________

4) Years passed since obtaining diploma/graduate degree

□ < 5 □ between 5 and 10 □ between 10 and 15 □ between 15 and 20 □ > 20 years

5) Years worked in the area where you are now?

□ < 5 □ between 5 and 10 □ between 10 and 15 □ between 15 and 20 □ > 20 years

SCHEDA DI RACCOLTA DATI DEMOGRAFICA e PROFESSIONALE (in italiano)

1) Sesso:

□ Maschio □ Femmina

2) Età:

□ < 25 anni □ dai 26 ai 30 □ dai 31 ai 35 □ dai 36 ai 40 □ dai 41 ai 45

□ dai 46 ai 50 □ dai 51 ai 55 □ > 55 anni.

3) Qualifica professionale:

□ infermiere □ infermiere pediatrico □ altro (specificare)_______________________________

4) Anni passati dal conseguimento del diploma/laurea di base

□ < 5 □ tra 5 e 10 □ tra 10 e 15 □ tra 15 e 20 □ > 20 anni

5) Anni di lavoro presso l’area in cui si trova ora?

□ < 5 □ tra 5 e 10 □ tra 10 e 15 □ tra 15 e 20 □ > 20 anni
