# Supplementary material for: Optimizing Workflow, Safety and Children’s Comfort in the Operating Theatre: A Mixed-Method Study Exploring Nurses’ and Caregivers’ Experiences and Possible Areas for Improvement
Source: Children (Basel). 2026 Apr 10;13(4):528. doi: 10.3390/children13040528 (PMC13115178; doi:10.3390/children13040528)
Supplement: Supplementary file 1 [file children-13-00528-s001.zip › Supplementary file S3. Questionnaire nurses' experiences.pdf]

Supplementary file S3. QUESTIONNAIRE: "NURSES EXPERIENCE WITH CAREGIVER PRESENCE CLOSE TO THE CHILD IN THE OPERATING THEATRE"

(In English)

Dear nurse,

we ask for your willingness to fill in this short questionnaire concerning your experience with the caregiver presence close to the child in the operating theatre. Your answers will help us optimize the presence of the caregiver in the operating theatre.

**Statements**	**Assign an "X" in the box corresponding to the numerical value your experience**				
Assign a value from 1= strongly disagree, to 5= strongly agree to the following statements	1 (strongly disagree)	2	3	4	5 (strongly agree)
1. The experience of the caregiver accompanying the child to the operating theatre was positive					
2. In the operating theatre the caregiver was anxious or agitated					
3. You noticed that the child was anxious or agitated					
4. You perceive that caregivers are timely informed about all matters concerning the operating theatre and the necessary support for the child in the operating theatre					
5. You think that caregivers need more information before accompanying the child to the operating theatre					
6. You perceive that caregivers experienced difficulties during the time in the operating theatre with the child					
7. You perceive that caregivers' support to the child in the operating theatre made him/her feel more comfortable					
8. You perceive that caregivers are comfortable in the operating theatre					
9. In the operating theatre caregivers are competent in supporting the child					
10. You think that caregivers need some additional advice/strategies to distract/relieve the child in the operating theatre					
11. In the operating theatre caregivers are distracted by the surroundings and are not able to give the child the attention needed					
12. Caregivers know how long they can stay with the child in the operating theatre					
13. You perceive that caregivers are adequately informed about what they can touch and not touch in the operating theatre					
14. You perceive that caregivers know that they have to wear a gown, cap and overshoes before					

entering the operating theatre					

(In Italiano)

QUESTIONARIO: "L'ESPERIENZA DEGLI INFERMIERI CON LA PRESENZA DEL CAREGIVER VICINO AL BAMBINO IN SALA OPERATORIA"

Gentile infermiera,
le chiediamo la disponibilità a compilare questo breve questionario sulla sua esperienza con la presenza dell'operatore vicino al bambino in sala operatoria. Le sue risposte ci aiuteranno a ottimizzare la presenza dell'assistente in sala operatoria.

**Affermazioni**	**Assegna una "X" nella casella corrispondente al valore numerico della tua esperienza**				
Assegna un valore da 1= fortemente in disaccordo, a 5= fortemente d'accordo alle seguenti affermazioni	1 (fortemente in disaccordo)	2	3	4	5 (fortemente d'accordo)
1. Consideri l'esperienza dei genitori che accompagnano il proprio figlio in sala come un'esperienza positiva per loro stessi					
2. In sala operatoria il genitore è in ansia o agitato/a					
3. In sala operatoria i bambini sono in ansia o agitati					
4. Ritieni che il genitore sia informato per tempo su tutte le questioni riguardanti la sala operatoria ed il supporto necessario per suo/a figlio/a in sala					
5. Pensi che il genitore debba ricevere più informazioni prima di accompagnare suo/a figlio/a in sala operatoria					
6. Il genitore incontra delle difficoltà nel tempo della sua permanenza in sala operatoria accanto a suo/a figlio/a					
7. Il supporto che il genitore da al proprio figlio/a in sala lo/a renda più tranquillo/a					
8. In sala operatoria, i genitori si sentono a proprio agio					
9. In sala operatoria, i genitori sono competenti nel supportare il loro figlio/a					
10. I genitori avrebbero bisogno di qualche ulteriore consiglio/strategia per distrarre/allentare la tensione del loro figlio/a in sala operatoria					
11. In sala operatoria il genitore è distratto dall'ambiente circostante e non riesce a dare tutta l'attenzione al loro figlio/a					
12. I genitori sanno fino a che momento possono rimanere accanto al loro figlio/a in sala operatoria					
13. I genitori sanno quello che possono toccare o non toccare in sala operatoria					

14. I genitori sanno di dover indossare camice, cuffia e sovrascarpe prima di entrare in sala operatoria					
