# Supplementary material for: Optimizing Workflow, Safety and Children’s Comfort in the Operating Theatre: A Mixed-Method Study Exploring Nurses’ and Caregivers’ Experiences and Possible Areas for Improvement
Source: Children (Basel). 2026 Apr 10;13(4):528. doi: 10.3390/children13040528 (PMC13115178; doi:10.3390/children13040528)
Supplement: Supplementary file 1 [file children-13-00528-s001.zip › Supplementary file S4. Questionnaire caregiver's experience.pdf]

Supplementary file S4. QUESTIONNAIRE "CAREGIVER EXPERIENCE IN THE OPERATING THEATRE"

(in English)

Dear parent/caregiver,

we ask for your willingness to fill in this short questionnaire concerning your experience in the operating theatre close to your son/daughter/child. Your answers will help us to make this journey more comfortable for your child and for you.

**Statements**	**Assign an "X" in the box corresponding to the numerical value your experience**				
Assign a value from 1= strongly disagree, to 5= strongly agree to the following statements	1 (strongly disagree)	2	3	4	5 (strongly agree)
1. Your experience of accompanying your son/daughter to the operating theatre was positive					
2. In the operating theatre you felt anxious or agitated					
3. You noticed that your child was anxious or agitated					
4. You consider that you have been timely informed about all matters concerning the operating theatre and the necessary support for the child in the operating theatre					
5. You would have liked to receive more information before accompanying your son/daughter to the operating theatre					
6. You experienced difficulties during your time in the operating theatre with your son/daughter					
7. You feel that the support you were able to give your son/daughter in the operating theatre made him/her feel more comfortable					
8. You were comfortable in the operating theatre					
9. In the operating theatre you felt competent to support your son/daughter					
10. you feel that you would have needed some additional advice/strategies to distract/relieve your son/daughter in the operating theatre					
11. In the operating theatre you were distracted by the surroundings and were not able to give your son/daughter the attention needed					
12. You knew how long you would be able to stay with your son/daughter in the operating theatre					
13. You felt adequately informed about what you could touch and not touch in the operating theatre					
14. You knew you had to wear a gown, cap and overshoes before entering the operating theatre					

(In Italiano)

QUESTIONARIO SULL'ESPERIENZA IN SALA OPERATORIA PER GENITORE/CAREGIVER

Gentile genitore/caregiver,
chiediamo la Sua disponibilità a compilare questo breve questionario riguardante l'esperienza in sala operatoria accanto a suo figlio/a, bambino/a. Ogni Sua risposta ci sarà utile per rendere questo percorso più confortevole per i vostri bimbi e per voi genitori.

**Affermazioni**	**Assegna una "X" nella casella corrispondente al valore numerico della tua esperienza**				
Assegna un valore da 1= fortemente in disaccordo, a 5= fortemente d'accordo alle seguenti affermazioni	1 (fortemente in disaccordo)	2	3	4	5 (fortemente d'accordo)
1. La sua esperienza nell'accompagnare suo figlio/a in sala operatoria è stata un'esperienza positiva					
2. In sala operatoria si è sentito in ansia o agitato/a					
3. Ha notato che suo figlio/a era in ansia o agitato/a in sala operatoria					
4. Ritiene di essere stato informato per tempo su tutte le questioni riguardanti la sala operatoria ed il supporto necessario per suo figlio/a in sala					
5. Avrebbe voluto ricevere più informazioni prima di accompagnare suo figlio in sala operatoria					
6. Ha incontrato delle difficoltà nel tempo della sua permanenza in sala operatoria accanto a suo figlio/a					
7. Ritiene che il supporto che è riuscito/a a dare a suo figlio/a in sala operatoria lo/a abbia reso/a più tranquillo/a					
8. In sala operatoria si è sentito/a a proprio agio					
9. In sala operatoria si è sentito competente nel supportare suo figlio/a					
10. Avrebbe avuto bisogno di qualche ulteriore consiglio/strategia per distrarre/allentare la tensione a suo/a figlio/a in sala operatoria					
11. In sala operatoria era distratto/a dall'ambiente circostante e non è riuscito a dare tutta l'attenzione necessaria					
12. Sapeva fino a che momento sarebbe potuto/a rimanere accanto a suo/a figlio/a in sala operatoria					
13. Si sentiva adeguatamente informato/a riguardo ciò che poteva toccare o non toccare in sala operatoria					
14. Sapeva di dover indossare camice, cuffia e sovrascarpe prima di entrare in sala operatoria					
