# Supplementary material for: Optimizing Workflow, Safety and Children’s Comfort in the Operating Theatre: A Mixed-Method Study Exploring Nurses’ and Caregivers’ Experiences and Possible Areas for Improvement
Source: Children (Basel). 2026 Apr 10;13(4):528. doi: 10.3390/children13040528 (PMC13115178; doi:10.3390/children13040528)
Supplement: Supplementary file 1 [file children-13-00528-s001.zip › Supplementary file S5. Sociodemographic for caregivers.pdf]

Supplementary file S5. SOCIODEMOGRAPHIC DATA COLLECTION SHEET FOR CAREGIVERS IN ENGLISH AND ITALIAN

SOCIODEMOGRAPHIC DATA COLLECTION SHEET FOR CAREGIVERS (In English)

1. How old are you? Years old:______________

2. Sex? □ Male □ Female

3. In what country were you born?
a. Italy
b. Altro (specificare)_______________

4. Your mother tongue is:
a. Italian
b. Altro (specificare)_______________

5. What is the highest level of schooling you have obtained?
a. None (not yet)
b. Primary school
c. Lower secondary Schools
d. Upper secondary schools
e. University (three-year/majors/specialist)
f. Post-university (master's/doctorate)
g. Other (specify)________________

6. What job do you currently do?
a. Manager/Employer
b. Self-employed
c. Employed
d. Worker
e. Not employed/unemployed
f. Other (specify) __________________

7. Are you a health professional (e.g. doctor, nurse, social worker...)? □ Yes □ No

8. Has the child/young adult already had other experiences with surgery in hospital?
□ Yes □ No

9. Relationship you have with the child:
□ is mother/dad □ guardian □ other (please specify) _________________________

10. How old is the child? Aged:______________

11. Sex of child? □ Male □ Female

12. What operation did the child undergo?
____

SCHEDA RACCOLTA DATI SOCIO-DEMOGRAFICA PER CAREGIVERS (in Italiano)

1. Quanti anni ha? Anni compiuti:______________

2. Sesso? □ Maschio □ Femmina

3. In che Paese è nato/a?
a. Italia
b. Altro (specificare)_______________

4. La sua lingua madre è:
a. Italiano
b. Altro (specificare)_______________

5. Qual è il livello più alto di scolarità da lei ottenuto?
a. Nessuno (non ancora)
b. Scuole elementari
c. Scuole medie inferiori
d. Scuole medie superiori
e. Università (triennale/magistrale/specialistica)
f. Post-università (master/dottorato)
g. Altro (specificare)________________

6. Quale lavoro fa attualmente?
a. Dirigente/Imprenditore
b. Libero professionista
c. Impiegato/a
d. Operaio/a
e. Non occupato/disoccupato
f. Altro__________________

7. E' un professionista sanitario (es. medico, infermiere, operatore socio sanitario…)?
□ Si □ No

8. Il/la bambino/a ha già avuto altre esperienze di interventi chirurgici in ospedale?
□ Si □ No

9. Relazione che ha con il/la bambino/a:
□ è mamma/papà □ tutore □ altro (specificare) __________________________

10. Quanti anni ha il/la bambino/a? Anni compiuti:______________

11. Genere del bambino/a? □ Maschio □ Femmina

12. Che operazione ha subito il /la bambino/a?
____
