# Supplementary material for: Optimizing Workflow, Safety and Children’s Comfort in the Operating Theatre: A Mixed-Method Study Exploring Nurses’ and Caregivers’ Experiences and Possible Areas for Improvement
Source: Children (Basel). 2026 Apr 10;13(4):528. doi: 10.3390/children13040528 (PMC13115178; doi:10.3390/children13040528)
Supplement: Supplementary file 1 [file children-13-00528-s001.zip › Supplementary file S6. Type of surgery.pdf]

Supplementary file S6. Type of surgery and classification on the base of recovery time.

**Type of surgery**	**Classification codes**
Screw removal from feet	1
Adenoidectomy	1
Chalazion removal	1
Adenotonsillectomy	1
Frenulectomy (upper lip and tongue)	1
Hypospadias repair	2
Oral cavity sanitation	1
Flat feet correction	1
Arm fracture treatment	1
Adenoidectomy + tympanic drainage	1
Scoliosis surgery	2
Ovarian cyst removal	2
Circumcision	1
Orchidopexy	1
Phimosis surgery	1
Inguinal hernia repair	1
Pyeloureteroplasty	2
Bilateral tendon lengthening	2
Strabismus correction	1
Bone cyst infiltration	1
Myringoplasty	1
Testicular detorsion	1
Instrumentation distraction	2
Tympanoplasty	1
Rectal reconstruction	2
Anorectoplasty	2
Knee surgery	1
Umbilical hernia repair	1
Costal distractor lengthening	2
Radial fracture treatment	1
Synthesis removal from malleolus	1

Hydrocele surgery	1
Valgus knee correction	1
Partial toenail removal	1
Revision tympanoplasty and ossiculoplasty	1
Clubfoot correction	1
Femur plate and screw removal	1
Cyst removal	1
Lacrimal duct probing	1
Varicocele surgery	1
Fistula closure	2
Short frenulum repair, preputial plastic surg.	1
Otoplasty	1
Hallux valgus correction	1
Hypospadias repair with left testicle descent	2
Corporoplasty	1
